# Estimating the environmental impacts of global lithium-ion battery supply chain: A temporal, geographical, and technological perspective

**DOI:** 10.1093/pnasnexus/pgad361

**Published:** 2023-11-28

**Authors:** Jorge A Llamas-Orozco, Fanran Meng, Gavin S Walker, Amir F N Abdul-Manan, Heather L MacLean, I Daniel Posen, Jon McKechnie

**Affiliations:** Sustainable Process Technologies Research Group, Department of Mechanical, Materials and Manufacturing Engineering, Faculty of Engineering, University of Nottingham, Nottingham NG7 2RD, UK; Department of Chemical & Biological Engineering, Faculty of Engineering, Sir Robert Hadfield Building, Mappin Street, Sheffield S1 3JD, UK; Sustainable Process Technologies Research Group, Department of Mechanical, Materials and Manufacturing Engineering, Faculty of Engineering, University of Nottingham, Nottingham NG7 2RD, UK; Strategic Transport Analysis Team, Transport Technology R&D, Research & Development Center (R&D), Saudi Aramco, Dhahran 31311, Saudi Arabia; Department of Civil & Mineral Engineering, University of Toronto, 35 St.George Street, Toronto, Ontario M5S 1A4, Canada; Department of Civil & Mineral Engineering, University of Toronto, 35 St.George Street, Toronto, Ontario M5S 1A4, Canada; Sustainable Process Technologies Research Group, Department of Mechanical, Materials and Manufacturing Engineering, Faculty of Engineering, University of Nottingham, Nottingham NG7 2RD, UK

**Keywords:** life cycle assessment, lithium-ion battery, supply chain GHG emissions, electricity decarbonization, battery recycling

## Abstract

A sustainable low-carbon transition via electric vehicles will require a comprehensive understanding of lithium-ion batteries’ global supply chain environmental impacts. Here, we analyze the cradle-to-gate energy use and greenhouse gas emissions of current and future nickel-manganese-cobalt and lithium-iron-phosphate battery technologies. We consider existing battery supply chains and future electricity grid decarbonization prospects for countries involved in material mining and battery production. Currently, around two-thirds of the total global emissions associated with battery production are highly concentrated in three countries as follows: China (45%), Indonesia (13%), and Australia (9%). On a unit basis, projected electricity grid decarbonization could reduce emissions of future battery production by up to 38% by 2050. An aggressive electric vehicle uptake scenario could result in cumulative emissions of 8.1 GtCO_2_eq by 2050 due to the manufacturing of nickel-based chemistries. However, a switch to lithium iron phosphate-based chemistry could enable emission savings of about 1.5 GtCO_2_eq. Secondary materials, via recycling, can help reduce primary supply requirements and alleviate the environmental burdens associated with the extraction and processing of materials from primary sources, where direct recycling offers the lowest impacts, followed by hydrometallurgical and pyrometallurgical, reducing greenhouse gas emissions by 61, 51, and 17%, respectively. This study can inform global and regional clean energy strategies to boost technology innovations, decarbonize the electricity grid, and optimize the global supply chain toward a net-zero future.

**Significance Statement**

Understanding the environmental impact of electric vehicle batteries is crucial for a low-carbon future. This study examined the energy use and emissions of current and future battery technologies using nickel-manganese-cobalt and lithium-iron-phosphate. We looked at the entire process from raw materials to battery production, considering emission reduction potential through cleaner electricity generation. We found that most emissions are concentrated in China, Indonesia, and Australia. By 2050, aggressive adoption of electric vehicles with nickel-based batteries could spike emissions to 8.1 GtCO_2_eq. However, using lithium iron phosphate batteries instead could save about 1.5 GtCO_2_eq. Further, recycling can reduce primary supply requirements and 17–61% of emissions. This study is vital for global clean energy strategies, technology innovation, and achieving a net-zero future.

## Introduction

To achieve a successful sustainable energy transition, the world will require significant volumes of metals and materials produced using low-carbon technologies. The push to electrify transport and the rise of battery electric vehicles (BEVs) will be key driving forces behind this growing demand for low-carbon materials (1). The global BEV fleet is expected to increase from 1.2 million in 2015 to 965 million in 2050, significantly boosting material demand for battery manufacturing (2). BEVs have zero tailpipe emissions, but they are not without environmental impact. Elsewhere in the global supply chain, greenhouse gas emissions are released, especially during the production of materials and battery manufacture. The mining and refining of materials, cell manufacturing, and battery assembly processes together account for 10–30% of the total life cycle emissions of a BEV (3). These negative externalities could potentially offset the absolute benefit of using BEVs to replace internal combustion engine vehicles (ICEVs). However, very high greenhouse gas (GHG) electricity intensity (almost entirely coal) would be required for a BEV to have higher life cycle emissions than ICEVs. Efforts to reduce the GHG intensity of energy use, particularly in electricity generation, could further reduce the emissions from BEVs in future. Emissions associated with BEV manufacturing (including batteries) will represent a larger portion of life cycle emissions when their use phase emissions are reduced due to charging with low GHG electricity. This study aims to quantify selected environmental impacts (specifically primary energy use and GHG emissions) of battery manufacture across the global value chain and their change over time to 2050 by considering country-specific electricity generation mixes around the different geographical locations throughout the battery supply chain.

Lithium-ion batteries (LIBs) are currently the leading energy storage systems in BEVs and are projected to grow significantly in the foreseeable future. They are composed of a cathode, usually containing a mix of lithium, nickel, cobalt, and manganese; an anode, made of graphite; and an electrolyte, comprised of lithium salts. Aluminum and copper are also major materials present in the pack components. The three main LIB cathode chemistries used in current BEVs are lithium nickel manganese cobalt oxide (NMC), lithium nickel cobalt aluminum oxide (NCA), and lithium iron phosphate (LFP). The most commonly used LIB today is NMC (4), a leading technology used in many BEVs such as the Nissan Leaf, Chevy Volt, and BMW i3, accounting for 71% of global battery sales (5, 6). NMC batteries are favored for their relatively high specific energy: Nickel improves the specific energy of NMC but at the expense of the battery's stability; on the other hand, manganese delivers good stability while compromising its specific energy (7). The NMC cathode chemistry comes in various commercial formulations, mainly NMC111, NMC622, and NMC811, where the numerical suffixes—111, 622, and 811—represent the molar proportions of nickel, manganese, and cobalt in the cells, respectively. There is a trend toward cathodes with higher nickel content as battery producers increasingly thrift on cobalt due to supply issues (8). The NCA cathode is commonly used in Tesla vehicles, while Volkswagen typically favors NMCs. However, with the expiry of the LFP patent in 2022 (9), major automotive manufacturers outside of China are showing interest in LFP batteries, particularly for entry-level high-volume BEVs, given their cost advantages (10, 11). LFP batteries are mostly used in BEVs in China, but battery productions in Europe and North America are likely to shift to LFP to meet projected demand growth. While LFP batteries have lower energy density than BEVs with nickel-based LIB chemistry, interest in LFPs is growing, mainly driven by their cost advantage. LFP is still exposed to rising lithium prices, but it does not contain nickel and cobalt, thus avoiding price and market volatilities typically associated with these commodities (12). Moreover, the latest cell-to-pack technology innovation could reportedly increase the energy density of an LFP to about 85% of that of an NMC811 battery (13).

Supply chains of LIB materials are characterized by highly global trade, with energy-intensive activities related to ore extraction, processing, and refining taking place across a wide range of locations globally (14). Key materials used in a LIB include nickel, cobalt, manganese, graphite, and lithium. According to Brown et al. (15), nickel is mined in more than 25 countries worldwide, however, Indonesia and Russia are the largest nickel producers with 38 and 11%, respectively; 63% of cobalt is extracted from the Democratic Republic of Congo; manganese is mainly mined in South Africa (30%) and Australia (12%); 62% of graphite is produced in China; and lithium is mainly mined in Australia (52%) and South America (Chile 22%, Argentina 7%). However, LIB refining and manufacturing are dominated by China. More than half of cobalt, graphite, and lithium refining capacity is situated in China and the country produces over 75% of all LIBs (16). Europe is responsible for ∼10% of global LIB manufacturing but is expected to increase its capacity to reach 25% by 2030. The United States has 6% of LIB production capacity, while Japan and South Korea together have 5%. China's dominance is likely to remain through 2030 (12). However, the geographical distribution of key battery materials is sufficiently diverse to require detailed consideration of the multiple locations where each material is mined and processed throughout the supply chain to derive the global-average climate change impact of LIBs. Global-average impacts are important because they reveal the current state of LIBs manufacturing, while keeping consistency with previous studies. However, detailed emission factors for specific supply chains become more important because they allow the quantification of current and future energy and GHG emissions of battery manufacturing. Therefore, we must consider specific locations where key activities are occurring today, how they will shift in the future and the evolution of battery technologies over time.

In the last decade, many life cycle assessment studies have assessed the cradle-to-gate environmental impacts of existing LIB technologies. The reported GHG emissions range from 39–196-kgCO_2_eq/kWh battery due to differences in many factors including battery specifications and technologies, geographical locations, and life cycle inventory data; and material, energy, and processes emission factors; making direct comparison of results very difficult (6, 17, 18). These studies widely report that electricity use accounts for the largest contribution (∼40%) to the overall life cycle GHG emissions from LIB manufacturing. This highlights the critical importance of decarbonizing the electricity sector as a key strategy to reduce overall GHG emissions. It is worth noting that emissions from electricity generation vary considerably between regions. Therefore, using a region-specific electricity mix is critical when assessing the energy use and GHG emissions of LIB manufacturing (6, 19). Several of these studies focus on individual countries. For instance, Kim et al. (17) focused on South Korea and found an emission intensity of 141-kgCO_2_eq/kWh battery. Sun et al. studied China and reported an emission intensity of 124.5 kgCO_2_eq/kWh (20), while Dai et al. examined the United States and found an emission intensity of 72.9-kgCO_2_eq/kWh battery (6). Therefore, while their findings are insightful, they cannot be generalized worldwide. This will be even more limiting when LIB production expands globally in the near future to meet the rising demand for BEVs. In the coming decades, the power sector must shift to more renewable electricity to be aligned with the 2°C target, which implies a lower amount of GHG emitted per kWh of electricity generation. It is well-known that a decarbonized electricity sector is important for reducing the life cycle GHG emissions from BEV use (21), but ultimately it can also drive down the overall emissions from LIB production and vehicle manufacturing.

Recycling poses great potential by recovering valuable battery materials to supply secondary materials and incorporating them into the battery supply chain (i.e. closed-loop recycling) (22). Secondary supply, via recycling from end-of-life batteries, can help reduce primary material supply requirements and alleviate the environmental burdens associated with the material mining and refining processes. However, although recycling offers useful complementary resources, it can only provide a small fraction of demand, meaning that recycling will not be able to cope with the expanding demand (23). The technical limitations of recycling are susceptible to the battery chemistry and the various recycling methods, which recover different materials at different rates and efficiencies (22, 24). The battery recycling capacity worldwide as of June 2021 is dominated by China with 80%, followed by South Korea 8.4% and the United States 7% (25). Europe is expected to develop a competitive and sustainable battery value chain in the near future (26, 27).

This study aims to address the following research questions: (i) What are the energy use and GHG emissions associated with LIB manufacturing and different battery chemistries? (ii) In which geographical locations are these energy use and GHG emissions expected to occur? (iii) How are the energy use and GHG emissions expected to change toward 2050 by decarbonizing the electricity sector and considering various battery technology scenarios? (iv) What are the GHG emission reductions of battery manufacture using secondary materials via different recycling technologies? These research questions are addressed by investigating the battery-related energy use and emissions in the future, the drivers of these energy use and emissions, and possible mitigation strategies. This approach looks into estimating future emissions, with detailed information into where in the world the emissions are occurring and different grid mix scenarios for many different countries at once. We explore the implications of decarbonizing the electricity sector over time, by adopting two scenarios from the IEA (Stated Policies Scenario, SPS, and Sustainable Development Scenario, SDS) and looking at different battery market shares to 2050, the variability of energy use and GHG emissions depending on source across the supply chain and the impact of battery recycling processes by assuming two scenarios (European Battery Scenario and Circular Battery Scenario).

## Results

This section explores the GHG emissions of different LIB technologies by looking at where in the world materials and battery manufacturing processes take place, the emissions associated with these activities, how these emissions are expected to change in the future by looking at the supply chain, and battery manufacturing emissions from secondary production. Thus, this section presents five assessments as follows: (i) total battery impacts, (ii) geographically explicit life cycle assessment (LCA) study of battery manufacturing supply chain, (iii) future impacts of battery manufacturing by decarbonizing the electricity sector to 2050, (iv) future impacts of battery manufacturing considering projected technology development and battery market share to 2050, and (v) closed-loop recycling and battery manufacturing using secondary materials. The results for energy use can be found in the supplementary information.

For simplicity, results and discussion focus primarily on NMC811 and LFP battery chemistries throughout the paper, but numerical data for all chemistries can be found in the supplementary information. NMC811 was selected because of its current global relevance in BEVs and its high energy density, while LFP is selected as it is expected to make up an increasingly important share of LIB in the market (28).

### Total battery production environmental impacts

Whole battery analysis reveals similar GHG emissions for all nickel-based chemistries ranging from ∼80 kgCO_2_eq/kWh (NMC111, NMC622, NMC811) to a maximum of 82 kgCO_2_eq/kWh (NCA). Detailed GHG and primary energy demand (PED) impacts for all chemistries are given in Table S11 in the supplementary information. Across all nickel-based battery chemistries, the manufacturing of the cathode, including the active material and cathode production process, contributes the largest GHG emissions share, accounting for nearly 60% of the total (cathode active material 44% and cathode production 12%). For the active materials, nickel production is GHG intensive, mainly due to the high electricity consumption of nickel mining in Indonesia (38.3%) and nickel refining in China (32.3%), and their correspondingly higher electricity GHG emission intensities (see Table S5 in supplementary information). In contrast, an LFP cathode has lower GHG emissions of around 17 kgCO_2_eq/kWh due to less reliance on GHG-intensive active materials. Detailed GHG emissions breakdown by material types for each LIB cathode is provided in Table S10 in the supplementary information. Apart from the active material, wrought aluminum, which is used as the current collector for the cathode electrode, as well as for the battery enclosure, contributes approximately 12% of the total emissions. The battery management system (BMS) or electronic components, while having a high energy demand in their production (505 MJ per kg of BMS; ∼29.39 kgCO_2_eq/kg of BMS), are only responsible for ∼2% of the total emissions per kWh of battery due to their minor share of battery material composition by weight (∼1.75%). Copper contributes the lowest GHG emissions: just over 1% of the total. In comparison, battery assembly is a significant source of emissions, representing about 21% of the total GHG emissions. Therefore, the location of the assembly plant is important due to variations in the electricity grid's GHG intensities.

**Fig. 1.**
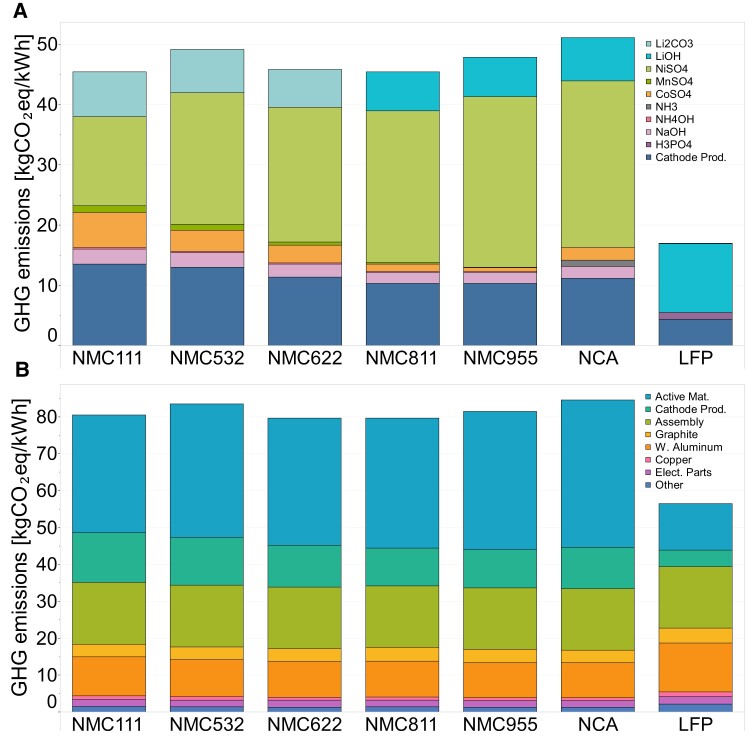
Cradle-to-gate GHG emissions of 1 kWh of different LIB technologies and the breakdown of contributions of the materials, along with the bill of material (BOM) (i.e. weights of different materials/components) and battery assembly. (A) Cathode active material. The cathode production process includes precursor co-precipitation and cathode production via calcination. (B) Total battery. Materials such as binder (polyvinylidene fluoride), electrolytes (LiPF6, ethylene carbonate, dimethyl carbonate), plastics (polypropylene, polyethylene, polyethylene terephthalate), steel, thermal insulation, and coolant are grouped into “Other” because they each contribute less than 1% to the total GHG emissions. Numerical data can be found in Tables S10 and S11 in the supplementary information.

The LFP battery has lower GHG emissions than any of the nickel-based chemistries, with an intensity of 55 kgCO_2_eq/kWh. This is due primarily to the lower impacts associated with cathode production. However, because of its lower energy density, an LFP battery is considerably bigger and heavier than nickel-based chemistry, which has about 20–40% higher gravimetric energy density. Therefore, other battery materials and the assembly process have a greater impact on an LFP battery than any of the nickel-based chemistries due to the lower energy density of the LFP chemistry and correspondingly greater battery size (see Figure S2 for PED figure). Figure 1 shows the cradle-to-gate GHG for 1 kWh of different LIB technologies.

### Supply chain environmental impacts (excluding recycling)

**Fig. 2.**
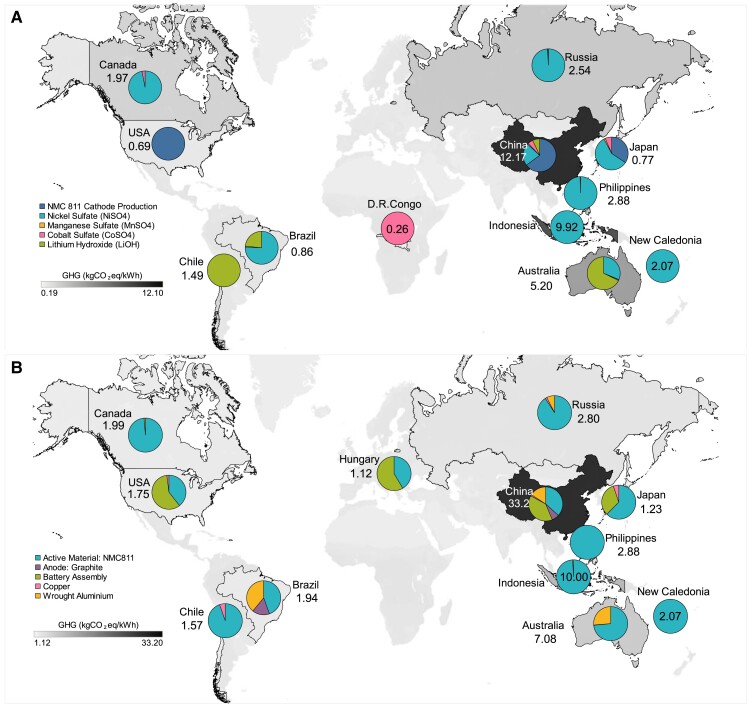
(A) Supply chain GHG emissions of the cathode active material (precursor) for NMC811 Li-ion battery—global production emissions of 45 kgCO_2_eq/kWh (B) supply chain GHG emissions of the total NMC811 battery—global-average production emissions of 79 kgCO_2_eq/kWh. Values on the map indicate the emissions in kgCO_2_eq/kWh battery. Detailed numerical values can be found in Tables S12 and S13, respectively, in the supplementary information.

**Fig. 3.**
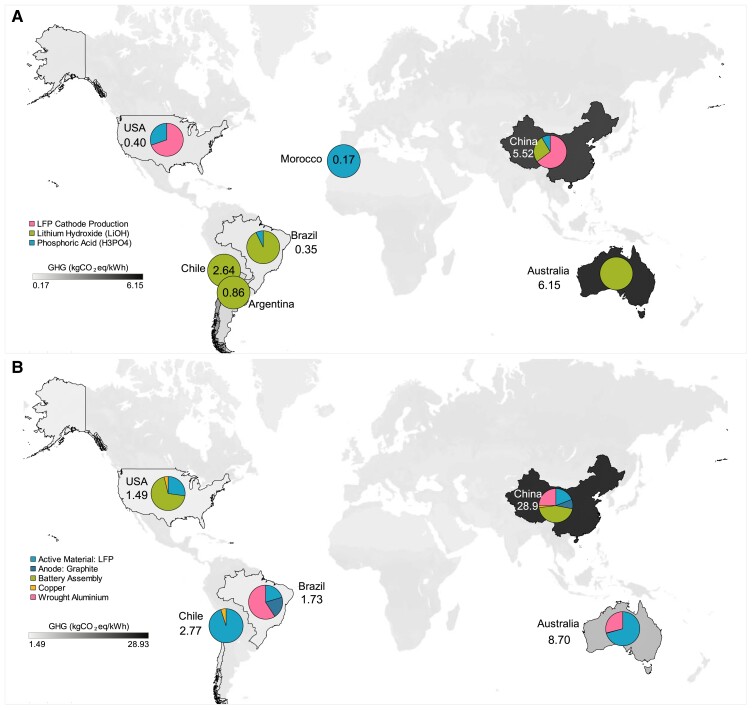
(A) Supply chain GHG emissions of the cathode active material for LFP Li-ion battery: global production emissions of 17 kgCO_2_eq/kWh (B) supply chain GHG emissions of the total LFP Li-ion battery production: global production emissions of 56 kgCO_2_eq/kWh. Values on the map indicate the emissions in kgCO_2_eq/kWh. Detailed numerical values can be found in Tables S14 and S15 in the supplementary information.

Globally, GHG emissions associated with LIB manufacture are concentrated in a small number of countries where material extraction, processing and refining, and battery manufacturing processes take place. Key drivers of GHG emissions include the production of nickel-based cathode materials, lithium, aluminum and graphite, as well as cathode manufacturing and battery assembly. Globally, GHG emissions hotspots relate to these key materials and LIB production activities. Global supply chain emissions for NMC811 cathode active material production and total battery production are shown in Figure 2A and Figure 2B, respectively; LFP cathode active material and total battery production are shown in Figure 3A and Figure 3B.

For the NMC811 cathode active material production and total battery production (Figure 2), global GHG emissions are highly concentrated in China, which represents 27% of cathode production and 45% of total battery production GHG emissions. As the world's largest battery producer (78% of global production), a significant share of cathode production and battery assembly occurs in China and these activities dominate China's contribution to the global GHG emissions of LIB manufacture. China is also a key nation for the refining of key battery materials. Although China does not possess an abundance of LIB deposits, it operates over 80% of global raw LIB material refining and is the world's largest producer of graphite, which is the primary anode material. With a fossil fuel-dominated electricity grid, China also has a GHG-intensive electricity mix (0.842 kgCO_2_eq/kWh) resulting in relatively high GHG emissions per unit of activity (29). Indonesia contributes the second largest share of NMC811 global total battery production emissions (13.5%) due to its large share of nickel mining and extraction activities (38%) and the highly emissions-intensive generation of 1.16 kgCO_2_eq/kWh of its electricity mix, which is also fossil fuel dominated (83%), with coal-fired generation representing 62.7% (29). In Figure 2, the value of 10 for Indonesia indicates its emission contribution in kgCO_2_eq/kWh battery. Australia contributes 9.5% to global emissions for NMC811 total battery production, due to its role in producing approximately half of the global lithium supply and significant nickel mining and refining operations. Detailed GHG emissions data by country are presented in supplementary information Table S12 (NMC811 active material) and Table S13 (total NMC811 battery). Emissions breakdown by countries for NMC811 and LFP cathode active material and total battery are portrayed in Figures S3 and S4, respectively.

Only a small number of countries represent significant contributions to the global supply chain GHG emissions of LFP batteries (Figure 3). As with NMC811, China dominates GHG emissions related to its dominating market share of cathode and battery manufacturing, as well as its role in refining key battery materials (lithium, aluminum, graphite, and copper). In total, 57% of LFP battery production emissions occur in China. Australia is the second greatest emissions source for LFP batteries due to its role in lithium and aluminum production, representing 17% of total emissions. Other countries that represent significant shares of LFP battery production are Chile (5%), Brazil (3%), and the United States of America (3%). Detailed GHG emissions data by country are presented in supplementary information Table S14 (LFP active material) and Table S15 (total LFP battery).

The total GHG emissions of LIB could be minimized by selecting material extraction, refining, and battery assembly locations with the lowest GHG emissions. For NMC811, this would entail mining nickel in Canada and refining in Norway; mining lithium in Brazil and refining it in the United States of America and assembling batteries in Hungary. This hypothetical scenario in 2020 would achieve life cycle GHG emissions of 57 kgCO_2_eq/kWh, a reduction of 26% compared to the current global-average production (77.4 kgCO_2_eq/kWh). Conversely, the greatest GHG emissions could be achieved by mining and refining nickel in Indonesia, mining and refining lithium in China, and assembling the battery in China, resulting in a total GHG emission of 85 kgCO_2_eq/kWh, an increase of more than 10% compared to the current global-average production. This suggests that there is considerable scope to reduce LIB production emissions by optimizing global supply chains, however, this can only happen with global governance on battery resources and manufacturing. Detailed sensitivity data of GHG emissions, considering the locations with maximum and minimum assessed emissions for each activity related to LIB production, are presented in Table S16 in the supplementary information, as well as in Figure S5.

### The key role of electricity decarbonization on future LIB production

**Fig. 4.**
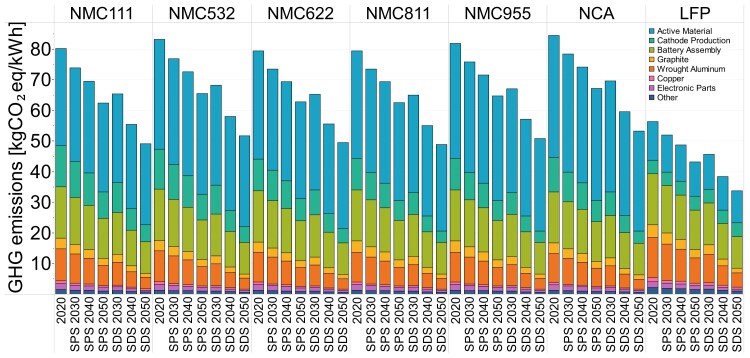
GHG emissions of LIB technologies considering the decarbonization of the electricity sector by 2050 in SPS and SDS scenarios. *y*-Axis indicates the GHG emissions in kgCO_2_eq/kWh battery, and *x*-axis indicates the decarbonization scenario by year.

Anticipated reductions in the GHG intensity of electricity generation reduce the life cycle GHG emissions of LIB manufacture toward 2050 in both scenarios as shown in Figure 4. Under the SPS scenario, life cycle GHG emissions of nickel-based batteries decline by 20–22% (from 77.4 to 61.7 kgCO_2_eq/kWh for NMC811, and from 82.3 to 66.4 kgCO_2_eq/kWh for NCA), primarily due to anticipated electricity sector decarbonization. This result is driven by reductions in the GHG intensity of wrought aluminum production (68%), battery assembly (38%), and cathode active material production (30%). Under the more ambitious SDS, GHG emissions would reduce by 37–39%. Similarly, for LFP, GHG emissions are reduced to ∼43 kgCO_2_eq/kWh (23% reduction) and ∼34 kgCO_2_eq/kWh (40% reduction), respectively, under the SPS and SDS scenarios in 2050. Some key materials see minor changes as a result of power sector decarbonization, such as nickel and lithium, with GHG emissions reducing to ∼19 and ∼5 kgCO_2_eq/kWh, a drop of 22 and 15%, respectively, under the SDS scenario. However, graphite and aluminum would experience substantial GHG emission reductions. Under the SDS scenario, graphite would reduce to about 1.4 kgCO_2_eq/kWh and aluminum to 3.4 kgCO_2_eq/kWh, a reduction of 59 and 63%, respectively. For the SPS scenario, GHG emissions of the cathode production process can be reduced to ∼6.1 kgCO_2_eq/kWh by 2050, a 37% reduction, and ∼3.7 kgCO_2_eq/kWh in the SDS (63% reduction). GHG emissions from battery assembly, led by China, would reduce by 21 and 36%, respectively, under the SDS scenario.

Electricity consumption contributes approximately 37% to the total current GHG emissions of LIB manufacture, so decarbonization of the electricity sector is an important lever in reducing the overall life cycle emissions. Non-electricity decarbonization inputs to material and battery manufacture are not considered in the present analysis, as there is generally greater uncertainty about the technological pathways and the timing of their deployments. This can include fuel switching potential and carbon capture and sequestration solutions.

A critical enabler for achieving these emission reductions for LIB manufacture is linked to China's power sector decarbonization, which is anticipated to lead to a significant reduction from 0.842 in 2020 to 0.078 in 2050 (kgCO_2_eq/kWh) under the SDS scenario, with total GHG emissions of 48.9 kgCO_2_eq/kWh battery (37% reduction). If a less aggressive GHG reduction is achieved by the electricity sector, e.g. 0.405 kgCO_2_eq/kWh, as under the SPS scenario, then a more modest reduction in LIB production emissions is realized (20% reduction) (see Table S17 and Table S18 in the supplementary information for detailed results for all scenarios). Importantly, there are other factors not included in the present analysis that could potentially have significant impacts on future LIB production emissions, positively and negatively. This includes reserve depletion (requiring energy-intensive extraction and processing of lower grade ores) and decarbonization of non-electricity energy inputs, such as fuels consumed by plant equipment and transport, and industrial heat. Opportunities for remanufacturing and recycling are limited in the near future as LIB capacity rapidly grows but will become more important as greater quantities of LIB reach their end of life; these factors are discussed in the next section.

### Anticipated reduced impacts with future battery technology mix

**Fig. 5.**
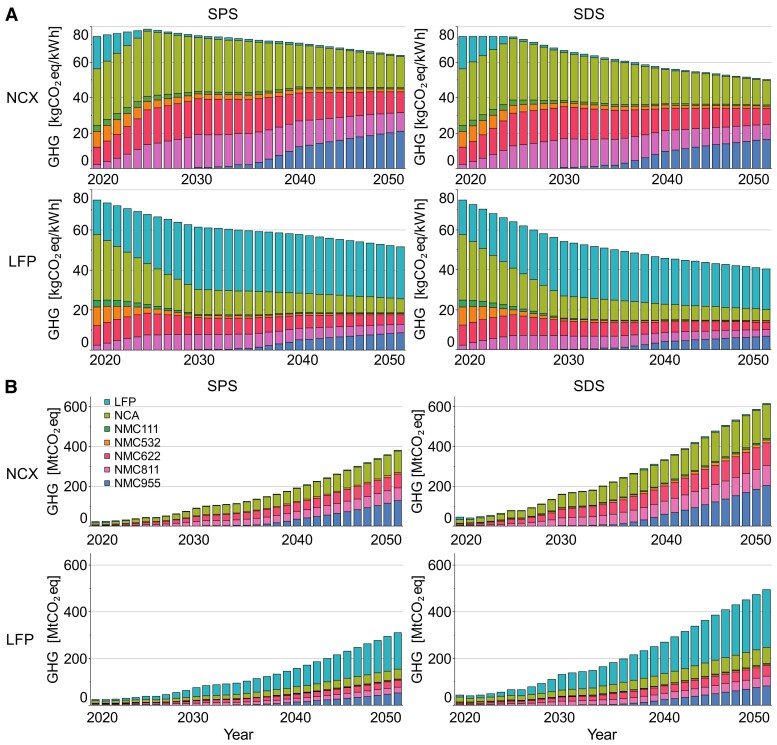
GHG emissions of LIB considering market shares by 2050 for the NCX and LFP battery scenarios coupled with the SPS and SDS electricity decarbonization scenarios. (A) Technology share-weighted projections of LIB GHG emission intensity to 2050—values in *y*-axis expressed in kgCO_2_eq/kWh (B) Annual and cumulative GHG emissions from battery manufacturing of different market shares and LIB demand scenarios, excluding battery replacement—values in *y*-axis expressed in MtCO_2_eq.

Technology share-weighted projections of LIB GHG emission intensity to 2050 are shown in Figure 5, indicating an overall reduction in the GHG intensity of LIB manufacture by up to nearly 50% by 2050 across the two scenarios considered. Projected future LIB GHG emissions are dependent on the assumed technology mix under both scenarios, as well as the depth of GHG reductions achieved in the electricity sectors of countries active in material and battery production. Reliance on nickel-based batteries (denoted NCX, where X indicates either Al or Mn) results in the highest LIB emissions of the scenarios considered. Initially, to 2025, a small increase in GHG emissions is anticipated in this scenario as LFP batteries are replaced by more GHG-intensive nickel-based alternatives, a driver that exceeds the near-term reduction in electricity GHG intensity. Average GHG emissions subsequently decline to reach a minimum value in 2050, driven by the anticipated reduction of GHG emissions in the electricity sector, representing a reduction of 13% compared to current emissions under the SPS scenario (63.3 kgCO_2_eq/kWh) and 30% under the SDS scenario (50.6 kgCO_2_eq/kWh). The LFP scenario sees immediate GHG emission reductions due to the near-term reduction in electricity GHG intensity and the longer-term replacement of nickel-based batteries with the lower GHG LFP alternative. The combination of these two factors results in the lowest projected LIB GHG emissions of the scenarios considered, with technology mix-weighted GHG emissions reducing to 51.1 kgCO_2_eq/kWh and 40.7 kgCO_2_/kWh (SPS and SDS scenarios, respectively). Detailed future battery emissions from different market shares can be found in Table S19 in the supplementary information.

Considering the anticipated scale of BEV deployment, decisions on LIB chemistry and electricity sector decarbonization have a significant influence on cumulative emissions to 2050. The IEA projects that total LIB capacity will exceed 12,000 GWh by 2050 under the SDS; primary manufacturing to create this battery capacity would result in GHG emissions totaling 8.2 GtCO_2_eq under the NCX scenario where nickel-based battery chemistries dominate. Achieving the same capacity under the LFP scenario would result in 1.5 GtCO_2_ fewer GHG emissions by 2050, a significant GHG emission savings equivalent to ∼3% of current global annual GHG emissions. These results are based on primary production only and do not consider a battery replacement. Battery replacement creates an opportunity for remanufacturing and recycling to reduce the GHG emissions burden compared to primary production, so in practice, the additional GHG emissions related to replacement can be less if a proper recycling ecosystem is in place. However, this must be balanced against declining ore quality that may drive up energy use, or the current trend toward larger battery sizes for longer range and/or larger (hence heavier) BEVs segments.

Interestingly, under the less aggressive decarbonization trajectory (i.e. SPS scenario), the cumulative GHG emissions for both LIB scenarios are lower than the SDS scenario. Although the grid is cleaner under the SDS scenario, the demand for LIB is projected to increase significantly by 2050 (12,000 GWh), almost doubling the LIB demand in 2050 under the SPS scenario (6,000 GWh). Detailed annual and cumulative GHG emissions associated with LIB manufacture under the set of future scenarios, as well as the battery capacity, are shown in Table S20.

### Environmental impacts of secondary battery materials from recycling

Future supply of secondary battery materials via end-of-life recycling can reduce reliance on primary materials—and associated GHG emissions—but the contribution is inherently limited by the mismatch of rapidly growing battery material demand and lesser availability of secondary materials. Recycling technologies vary in their ability to recover LIB materials, which will further influence the potential contribution of secondary materials to future LIB manufacture and their associated GHG emissions. This study assumes two recycling scenarios, firstly based on meeting the mandatory minimum levels of recycled content regulated by the EU (27) (“European Battery Scenario”) with assumed usage rates for other key battery materials (aluminum, copper). A second scenario (“Circular Battery Scenario”) considers the potential contribution of secondary materials supply if battery demand were to approach an equilibrium and manufacture needs only to replace retiring capacity, with closed-loop recycling providing a much greater share of LIB material demand. This second scenario is not intended to provide a realistic prediction. Instead, it serves as an illustrative scenario to better understand the limitations of LIB recycling in meeting future material demands, especially considering limited growth in the LIB market.

EU-mandated minimum recycled content in LIBs of 20% cobalt, 12% nickel, and 10% lithium and manganese will contribute to reducing associated GHG emissions by 7 to 42% for NCX chemistries. Among the different recycling methods, direct recycling has the lowest impact, followed by hydrometallurgical and pyrometallurgical. Pyrometallurgical recycling recovers only cobalt, nickel, and copper, and so alone is unable to meet the minimum secondary lithium content requirement. This recycling route results in the highest reliance on primary materials as a consequence, which also corresponds to the smallest reduction in GHG emissions relative to primary production (7–20% for NCX). In contrast, hydrometallurgical recycling can recover all key cathode and anode materials, including lithium, and so can achieve deeper GHG emission reductions by avoiding a greater fraction of primary material demand (11–25% for NCX). Direct recycling offers the lowest impact by physically separating battery components (graphite, aluminum, copper) and recovering the functional cathode structure without decomposition into substituent elements, hence accomplishing greater GHG emission reductions (37–42% for NCX). GHG emissions for secondary nickel-based cathode chemistries are in line with primary nickel-based chemistries, i.e. GHG emissions are dominated by the active materials (predominantly nickel, lithium/carbonate hydroxide, and cobalt for cobalt-rich chemistries) and the cathode production process. Although the BMS or electronic components are not included in the recycling process due to a minor share of emissions for primary production, there is an opportunity for recycling electronic waste.

Under the EU Battery Scenario, recycling methods in Europe yield varying GHG emissions reductions, with pyrometallurgical recycling reducing emissions by 4–18%, while hydrometallurgical and direct recycling achieve deeper reductions (8–22% and 36–41%, respectively). In the Circular Battery Scenario, relying heavily on secondary supply, GHG emissions reductions are significantly higher, ranging from 11–21% for pyrometallurgical, 49–54% for hydrometallurgical, to 55–63% for direct recycling. Notably, the intensity of electricity emissions during recycling plays a crucial role, being considerably lower (0.335 gCO_2_eq/kWh for Europe) than primary production from a mix of countries (0.838 gCO_2_eq/kWh).

Recycling efforts in China and the US under EU regulations yield smaller emissions reductions compared to Europe. A similar trend is observed within Europe, with the average EU mix serving as a middle ground in emissions reductions between individual countries like Germany and the UK. Despite challenges, recycling electronic waste presents an additional opportunity to further mitigate GHG emissions.

**Fig. 6.**
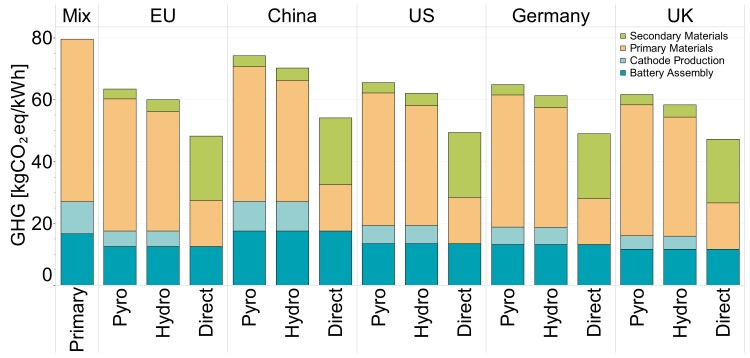
Primary NMC811 battery production GHG emissions compared to GHG emissions from secondary materials, cathode production, and battery assembly from pyrometallurgical, hydrometallurgical, and direct recycling technologies using electricity grid from Europe's average, China, United States, Germany, and United Kingdom, under the EU battery recycling scenario. Mix means the mix of countries containing the global-average GHG emissions assessed in previous sections.

Figure 6 shows the GHG emissions resulting from primary materials, secondary materials, cathode production, and battery assembly from pyrometallurgical, hydrometallurgical, and direct recycling technologies using electricity grid from Europe's average, China, United States, Germany, and United Kingdom, under the EU battery recycling scenario. The recycling of transition materials (including lithium, nickel, and cobalt) in the active cathode material and the recycling location provide the largest GHG emission reductions. Detailed numerical data for all GHG and PED under both scenarios can be found in Tables S26 and S27 in the supplementary information.

### The key role of electricity decarbonization coupled with future LIB recycling

**Fig. 7.**
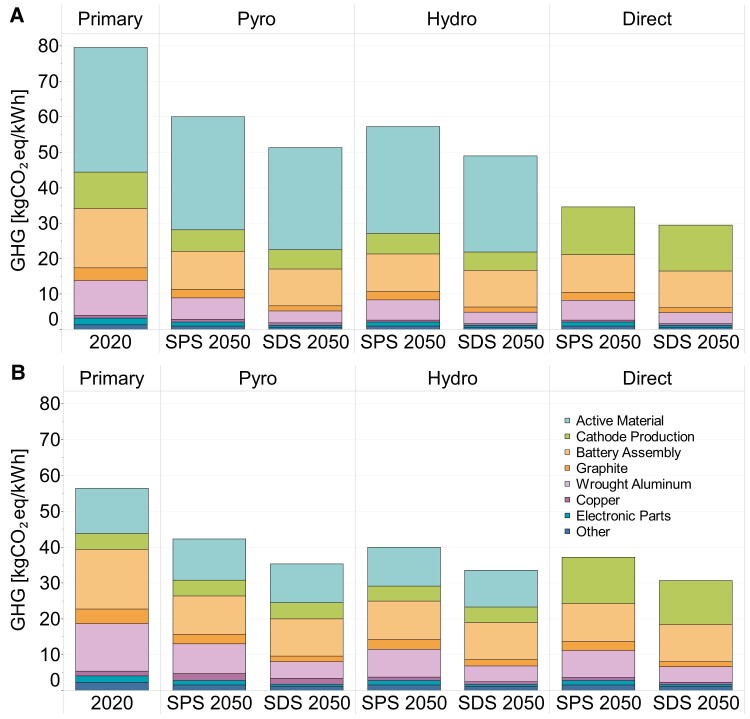
GHG emissions of (A) NMC811 and (B) LFP battery chemistry considering the decarbonization of the electricity sector to 2050 in SPS and SDS scenarios for pyrometallurgical, hydrometallurgical, and direct recycling technologies under the European Battery Scenario.

Projected decarbonization in the European electricity grid favors the battery recycling processes and thus the life cycle GHG emissions of battery manufacturing to 2050. For simplicity, the subsequent results discussion focuses on the European Battery Scenario for the SPS and SDS decarbonization scenarios to 2050; results for the Circular Recycling Scenario can be found in the supplementary information (S28). Figure 7 shows the GHG emissions of NMC811 and LFP battery production from pyrometallurgical, hydrometallurgical, and direct recycling technologies under European Battery Scenario for both decarbonization scenarios to 2050. Under the SPS scenario, the GHG emissions of the NMC811 can be reduced by 22%, 26%, and 55% for pyrometallurgical, hydrometallurgical, and direct recycling, respectively, compared to primary production of 77.4 kgCO_2_eq/kWh. This is mainly driven by reductions in the GHG intensity of several material production including the active material (70%), wrought aluminum (43%), graphite (38%), and copper (35%). Under the SDS scenario, various key materials see significant changes as a result of electricity decarbonization coupled with recycling. Via hydrometallurgical recycling, GHG emissions of nickel and lithium are 18.4 and 4.8 kgCO_2_eq/kWh, a reduction of 26 and 25%, respectively. GHG emissions of the cathode production process can be reduced to around 2.4 kgCO_2_eq/kWh, over a 65% reduction. Battery assembly, assuming to take place in Europe, would reduce by 38% under the SDS scenario.

Europe's electricity decarbonization is on track to meeting climate goals, thus contributing to a significant GHG reduction in battery production. Europe's GHG electricity intensity would see a reduction from 0.336 kgCO_2_eq/kWh in 2020 to 0.081 kgCO_2_eq/kWh of electricity in 2050, under the SDS scenario.

Moreover, GHG emissions for the whole NMC811 battery production would reduce to 51.3 kgCO_2_eq/kWh (34% reduction) for pyrometallurgical recycling, 49 kgCO_2_eq/kWh (37% reduction) for hydrometallurgical recycling, and 29.4 kgCO_2_eq/kWh (62% reduction) for direct recycling. The latter is 26.3 kgCO_2_eq/kWh less compared to decarbonizing only the electricity of NMC811 battery production in SDS by 2050. This points out the potential environmental benefits of recycling coupled with a less intensive grid. For LFP battery production, via direct recycling, GHG emissions can be reduced to 37.2 kgCO_2_eq/kWh (32% reduction) and 30.7 kgCO_2_eq/kWh (44% reduction), respectively, under the SPS and SDS scenarios to 2050.

If a less ambitious GHG reduction is achieved in the electricity sector under the SPS scenario, e.g., 0.109 kgCO_2_eq/kWh, then a moderate reduction in LIB production emissions would be achieved: 60 kgCO_2_eq/kWh for pyrometallurgical, 57.2 kgCO_2_eq/kWh for hydrometallurgical, and 34.5 kgCO_2_eq/kWh for direct recycling, respectively. Detailed numerical data for all chemistries and different recycling technologies under the different recycling and decarbonization scenarios can be found in Table S28 in the supplementary information.

## Discussion

Given the global decarbonization challenge and the scaled-up production required for LIBs, it is critical that the environmental impacts of LIB technologies are properly understood. In this study, the current and future life cycle environmental impacts of LIB manufacture are characterized spatially and temporally to better understand the role of electricity decarbonization and battery technology in a globalized LIB supply chain. It is demonstrated how GHG emissions can be shifted globally for a technology that is aimed at addressing a national GHG emission target (e.g. developed countries, mainly in the west, target BEVs to achieve 100% of new car sales as part of their national targets, GHG emissions from LIB could increase elsewhere, especially in the developing world in the east). Currently, China dominates the downstream battery supply chain, accounting for the largest share of supply chain GHG emissions, followed by Australia and Indonesia, depending on the battery technology type. However, this may change as LIB manufacturers emerge in different regions, and it is crucial that decisions on LIB productions also consider the overall life cycle emissions to minimize supply chain emissions.

To achieve a net-zero future, it is key to track upstream GHG emissions, or scope 3 emissions, throughout a global supply chain, and to identify measures to reduce them. It is found that decarbonizing electricity generation could substantially reduce battery production emissions toward 2050 as electricity consumption contributes approximately 37% of the total GHG emissions of LIB manufacture today. This analysis did not consider non-electricity inputs such as industrial heat and offroad mining that could potentially further reduce emissions by adopting low-carbon fuels and improving energy efficiency throughout the different life cycle stages of the battery. While there are opportunities to decarbonize these sectors, uncertainties surrounding the timeline and location of such efforts make it challenging to model these scenarios confidently. However, there is more clarity and planning regarding decarbonizing the electricity mix, providing a clearer understanding of future developments in the electricity sector compared to other excluded energy and material inputs in this analysis.

Deciding whether to shift battery production away from locations with emission-intensive electric grids, despite lower costs, involves a challenging balancing act. On the one hand, relocating to cleaner energy sources can significantly reduce the environmental impact of GHG emission-intensive battery production process (6, 14). On the other hand, this often comes at the expense of higher production costs, potentially affecting the batteries' competitiveness in the market (30). Policymakers and industry stakeholders must carefully weigh these environmental and economic factors while considering regional energy strategies and supply chain complexities. Investing in cleaner energy technologies and decarbonization efforts in existing locations can be alternative approaches to mitigate environmental concerns without immediate relocation. For example, recent announcements revealed that Tesla will build a new assembly plant in the north of Mexico, a region with water scarcity and an emission-intensive electric grid but with affordable and highly skilled labor, closeness to the United States and international free trade agreements (31–33). This new battery plant could incentivise the government to seek cleaner energy and support companies to meet their carbon neutrality pledges. Ultimately, the decision should align with broader sustainability goals and regional energy transition strategies while acknowledging the complexities of the battery industry’s global supply chain.

Companies choose the locations for battery plants based on various factors, including supply chain proximity, labor costs, regulatory environment, market access, carbon footprint, economic incentives, and commodity prices (34). Increasing battery demand might add supply issues to lithium, cobalt, and other raw materials. Some original equipment manufacturers (OEMs) aim to reduce emissions to 20 kg CO_2_e/kWh. In some instances, it could be feasible to reduce emissions by 80% with only a minimal increase in final costs (30). To achieve this, manufacturers must not only focus on using less emission-intensive electricity but also influence suppliers within the value chain to align with their sustainability goals.

Technology share-weighted projections of LIB to 2050 indicate an overall reduction in the GHG intensity of LIB manufacture of up to around 50% by 2050 across the two scenarios considering different technology mixes. However, it is important to note that the analysis is limited to the battery chemistries for which there is available data for modeling. Promising breakthrough battery chemistries like lithium-sulfur, lithium-silicon, lithium-air, solid-state, and sodium-ion batteries are not included in this analysis. This is due to their lack of commercial availability and limited data on material inventory and performance. As a result, their potential impact on GHG emissions and energy intensity in LIB manufacturing is not considered at this time. The depth of GHG reductions achieved in both scenarios also depends on the decarbonization rate of the electricity sector in countries active in the material and battery production supply chain.

Generally, secondary supply, via recycling, can help reduce primary supply requirements and alleviate the environmental burdens associated with the extraction and processing of materials from primary sources. The recycling scenarios assumed in this study are not meant as a prediction of what will happen but to contextualize how recycling could reduce impacts in the future. Therefore, these recycling scenarios look at the range of potential outcomes, comparing production from all materials with a maximum secondary material scenario (Circular Battery Scenario) and a more realistic scenario based on the mandatory minimum levels of recycled content declared by the new EU regulatory framework for batteries (European Battery Scenario) (27). The recycling scenarios are hypothetical since secondary materials will not be able to meet the rising demand for batteries, meaning that although recycling provides useful complementary resources, it can only provide a tiny fraction of total demand (23). However, this provides a holistic view of the possible outcomes of recycling, where direct recycling offers the lowest impacts, followed by hydrometallurgical and pyrometallurgical, reducing GHG emissions by 61, 51, and 17%, respectively, under the Circular Battery Recycling scenario, and 39%, 19%, and 15%, under the European Battery Scenario.

At the moment, China is the global leader in the battery recycling, accounting for 80% of the global capacity, and although this trend is expected to continue, the battery recycling landscape can evolve rapidly, and new developments in relevant regions such as North America, the European Union, South Korea, and Japan are likely to play a significant role in the future. The analysis shows a scenario where batteries are assumed to be recycled at different locations with distinct electricity mix. China, whose grid is still heavily coal-dependent, alleviates emissions by a lesser extent than if the recycling facility is located in a region/country with a less emission-intensive grid, such as the United Kingdom.

This study highlights the importance of having reliable primary data on material and energy consumption to quantify the supply chain environmental impacts associated with different LIB technologies. Future work should evaluate the role of other decarbonization measures involving non-electricity energy inputs and the potential for battery remanufacture/recycling/repurposing legislations in enabling a low-carbon and circular LIB economy. The evolution of LIB materials, innovation in breakthrough production and recycling technologies, and the optimization of global supply chains will be important changes that could impact the sustainability of LIBs in the foreseeable future (35).

### Recommendations

Decarbonizing the battery supply chain is crucial for promoting net-zero emissions and mitigating the environmental impacts of battery production across its lifecycle stages. The industry should ensure sustainable mining and responsible sourcing of raw materials used in batteries, such as lithium, cobalt, and nickel. By encouraging transparency of data throughout the supply chain, the overall carbon footprint of battery materials could be minimized, while promoting initiatives for ethical mining practices. The transition toward a cleaner electricity grid in battery manufacturing facilities can improve the overall environmental performance of battery production, however, additional efforts to improve energy efficiency and decarbonize non-electricity energy inputs are essential to reduce energy consumption and lower GHG emissions. The implementation of recycling programs and circular economy principles will ensure proper disposal and recovery of materials at the end of a battery's life cycle, while encouraging remanufacturing and reuse of secondary materials via recycling. In conclusion, this work highlights the importance of decarbonizing battery global supply chain, decarbonizing the electricity sector and the benefits of recycling to encourage a future sustainable battery ecosystem.

## Materials and methods

**Fig. 8.**
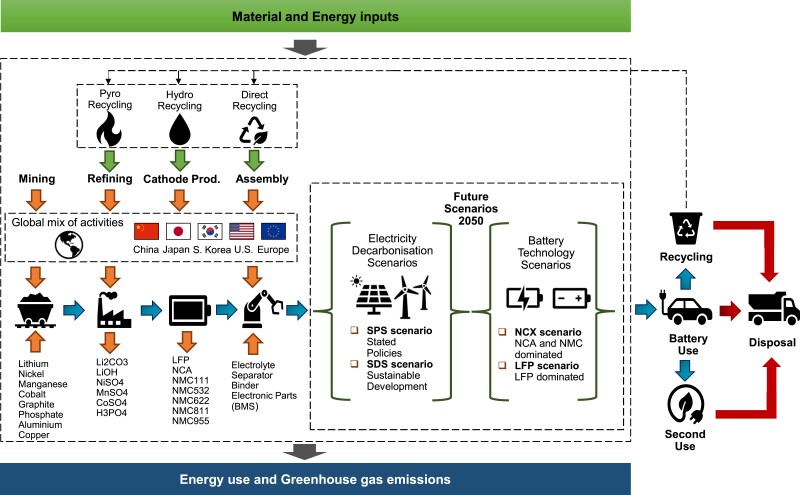
System boundary flowchart for battery chemistries and future scenarios to 2050. BEV, battery electric vehicle; Li_2_CO_3_, lithium carbonate; LiOH, lithium hydroxide; NiSO_4_, nickel sulfate; MnSO_4_, manganese sulfate; CoSO_4_, cobalt sulfate; H_3_PO_4_, phosphoric acid; NMC, lithium nickel manganese cobalt oxide; NCA, lithium nickel cobalt aluminum oxide; LFP, lithium iron phosphate; NCX, nickel cobalt (X denotes either Al or Mn—NCA or NMC).

This study presents a novel framework for the evaluation of life cycle environmental impacts (specifically, primary energy use and GHG emissions) of LIB manufacture and battery recycling. It provides spatial and temporal resolution to quantify where in the global supply chain GHG emissions occur and how these emissions are expected to vary over time (2020 to 2050) due to the anticipated uptake of competing battery chemistries and the decarbonization of the electricity sector as displayed in Figure 8. Life cycle inventories for LIB materials and manufacturing activities are coupled with data on the location of activities and location-specific emission factors for energy and material inputs to assess the global GHG emission implications of battery manufacture. A forward-looking analysis considers how the mix of LIB chemistries and background energy systems will drive future GHG emission trends to 2050. Finally, recovered materials from spent batteries are incorporated back into the battery supply chain (i.e. closed-loop recycling). Table S1 in the supplementary information provides a summary table that outlines the types of batteries considered, key materials and sources of data scenarios.

As shown in Figure 8, the system boundary accounts for all the battery production stages and their impacts associated with raw material extractions, (i.e. cradle), through to the assembly of the finished battery pack (i.e. gate). It does not include the downstream use phase but assesses end-of-life and closed-loop recycling. Across the LCA study, two environmental impact categories are considered since they are of general interest, relevant to climate change, and pertinent for LIBs. The selected categories are PED, i.e. the cumulative energy use associated with the production processes including fossil and renewable energy (MJ); and GHG emissions (i.e. CO_2_, CH_4_, and N_2_O), i.e. calculated based on 100-year global warming potentials i.e. as listed in the Fifth Assessment Report of the Intergovernmental Panel on Climate Change (36), expressed in kilograms of CO_2_ equivalent (kgCO_2_eq). The functional unit is 1 kWh of the battery pack for a passenger BEV.

The production processes of key battery materials, such as mining and refining, as well as battery manufacturing, are widely recognized as energy intensive. Therefore, the associated GHG emissions are primarily influenced by the energy sources used, with electricity being a significant contributor to the overall life cycle emissions. Therefore, this study focuses on analyzing the electricity mixes in different geographical locations along the battery supply chain, taking into account each country’s involvement in raw material mining or refining. This approach allows for an examination of the future electricity mixes in all countries within the supply chain. It is important to note that this study does not consider non-electricity inputs. Additionally, the analysis specifically focuses on selected battery chemistries (NMC, NCA, LFP) for both current and future scenarios, while other potentially promising battery chemistries can be included in future work.

Transportation emissions, while acknowledged, represent a relatively small proportion of total battery emissions (3–5%), as supported by literature (17, 30). This analysis incorporates default values for transportation emission across the mining to battery components production from the GREET model. A detailed analysis of transportation emissions falls outside the study's scope but could be explored in future research.

### Battery chemistries, materials inventories, and battery assembly

Battery chemistries relevant to current and future BEV applications are selected for the analysis, including as follows:

- NMC of varying compositions as follows: NMC111, NMC532, NMC622, NMC811, and NMC955;
- NCA; and
- LFP.

For each battery chemistry, the study integrates upstream material inventory data from GREET 2021 (37), which is assumed to be representative of current and future battery manufacture, to be consistent with GREET and previous studies to allow further comparisons. Detailed materials inventories for all chemistries are found in Table S2 in the supplementary information, which also provides additional spatial and temporal detail for the assessed materials (see Sections 2.2 and 2.3). Key battery materials are defined as those contributing more than 2% of total manufacturing emissions for one battery, as given by the default GREET analysis. Battery capacity is assumed to be 84 kWh, as provided by GREET as the default value.

LIB manufacturers are transitioning toward lower-cobalt cathodes, which has led to an evolution from NMC111 to NMC523, NMC622, NMC811, and, more recently, NMC955 which is expected to be available by 2030 (38, 39). However, LFP batteries are also being considered favorably for BEVs given their relatively low material cost and high abundance, e.g. Tesla, recently announced the use of LFP batteries in its Model 3 (10). Therefore, we set two main scenarios with varying market shares based on the assumed technological progress by (40) (a plot can be found in Figure S7 in the supplementary information) as follows:

- NCX scenario (X denotes either Al or Mn): Nickel and cobalt-containing batteries dominate the market by 2050. NMC955 is launched in 2030 and progressively substitutes for other NMC chemistries until achieving a third of the global market share by 2050. The shares of NCA and LFP chemistries reduce from 2030 at a similar rate.
- LFP scenario: The market share of LFP is assumed to increase steadily from 30% in 2020 to 60% by 2030 and remain constant until 2050. Non-LFP batteries lose market share proportionally, similarly to the NCX scenario.

Global battery manufacturing is projected to balloon this decade. In 2021, the Asia Pacific region, led by China, accounted for 84% of the global LIB manufacturing in 2021. China had a production capacity of 558 GWh (79% of the world total), the United States of America has 44 GWh (6% of the world total), and Europe had 68 GWh (9.6% of the world total) (16). Battery cell companies and startups have announced plans to build a production capacity of up to 2,357 GWh by 2030 (41). The growing sales of BEVs in China drive the country to lead the global LIB market capacity. China is projected to lead the market by 2030 with 1,247 GWh (53% world total), while the United States of America is set to produce 266 GWh (11.3% world total), and Europe 618 GWh (23.6% world total) (41). Germany will dominate Europe's LIB market capacity by 2030 with 266 GWh (10.4% world total), followed by France with 82 GWh (3.48% world total), the United Kingdom with 73 GWh (3.10% world total), and Poland with 68 GWh (2.89% world total) (41). Overall, the global LIB capacity could rise to around ∼6 TWh in the SPS and up to ∼12 TWh in the SDS by 2050 (40). This analysis assumes that the battery assembly market share stays constant after 2030, but the installed capacity follows the IEA's projections for 2050. Detailed projected battery assembly share mix by country and region is presented in Table S8 in the supplementary information.

### Global battery material production

This study analyzes the global differences in manufacturing processes and supply chain of battery materials by considering the mining and refining production shares of each country, along with their specific electricity mixes. The material production model is developed using the life cycle inventory in GREET 2021 (37) for key battery materials (see Section 2.1), extended to include a greater number of countries that are active in the mining and refining of key battery materials (responsible for more than 2% of mining or refining activity for each material). This is a wider reach than the GREET 2021 model (47 countries assessed in total). Mining, refining, and production data for key battery materials are obtained primarily from BGS World Mineral Production (15) and complemented with other relevant sources (42, 43). Detailed mining and refining data for key material production by country are presented in Table S3 in the supplementary information. The life cycle inventory, i.e. quantities of materials, processes direct energy, and non-electricity emission factors, is assumed to be unchanged for different production locations. Location-specific GHG emissions are assessed to account for differences in the country-specific electricity generation mix.

### Current and future electricity generation mixes

Location-specific electricity supply mixes, and resulting GHG intensities, are assessed for all countries identified as being significantly involved in the mining and refining of battery materials and battery assembly. Current country-specific electricity generation mixes are assessed using available data from IEA website (29), and electric power transmission and distribution data from World Bank (44). The PED and GHG emission impacts for 1 kWh of electricity generated in each country were calculated as the supply share-weighted average value, based on the ecoinvent database version 3.7 (45). Detailed inventory data of electricity for all relevant countries in the LIB production supply chain (including transmission and distribution losses) are listed in Table S4 and Table S5 in the supplementary information.

Two main IEA scenarios are considered to assess future electricity generation toward 2050 based on bottom-up electricity data as follows: the SPS and the SDS from the World Energy Outlooks (WEO) 2020 and 2021 (46, 47).

- The SPS reflects the effects of current policy frameworks and existing policy ambitions on the energy sector toward 2050. This scenario explores where the energy system might go without additional policy implementation (48). It provides a very ambitious, positive perspective toward achieving the Sustainable Development Goals (SDGs) related to energy (SDG7), environmental impacts (SDG3), and climate change (SDG13).
- The SDS is a “well below 2°C” pathway to achieve the climate goals agreed upon by the Paris Agreement as well as increasing the renewable energy integration and dramatically reducing the GHG emissions (49). In the SDS, many of the world's advanced economies reach net-zero emissions by 2050, China around 2060, and all other countries by 2070 (47).

Utilizing these scenarios for simulations, particularly in the context of electricity sector decarbonization, enables the exploration of diverse future pathways, assesses risks, and tests the effectiveness of policies and investments. Simulations facilitate long-term planning, resource allocation, and adaptation to uncertainties. The uncertainties surrounding scenarios like the SPS and the SDS are multifaceted and dynamic. They stem from unpredictable changes in energy policies, evolving technological landscapes, economic fluctuations, shifts in consumer and industry behavior, geopolitical events, uncertain responses to environmental and climate challenges, market dynamics, resource availability, and unforeseen disruptive events. These uncertainties highlight the need for flexible and adaptable strategies that can withstand a wide range of possible futures and underscore the importance of ongoing scenario planning. Regular updates and revisions to scenarios are crucial to account for emerging information and evolving circumstances.

The electricity mix scenarios focus on the 2020–50 generation. Each country's PED and GHG emissions of electricity generation are modeled based on the SPS and SDS scenarios. Where specific electricity generation data for countries were not available, generic IEA average world data were considered (29). Detailed future electricity mix shares and emission factors for all countries are listed in Tables S6 and S7, respectively (46, 47). Current and future country-specific GHG emissions are assessed for the mining and refining of battery materials (15, 42, 43), and battery assembly (16, 41, 50, 51), using Eq. 1 and portrayed in Figures 2 and 3 as follows:


(1) $$\mathrm{GHG}=\mathrm{NE}+\underset{i}{\sum }\;(E{}^{*}EF_{i}){}^{*}S_{i}$$

where the GHG generated is in kgCO_2_/kg material; NE is the GHG emissions arising from non-electricity inputs in kgCO_2_eq/kg material (as provided in Table S9 in the supplementary information), *E* is the electricity input in kWh per kg material, EF*_i_* is the emission factor of electricity intensity (kgCO_2_eq/kWh), and *S_i_* is the supply share of country *i* (in percentage unit).

### Battery recycling

The battery recycling model developed in this study uses data from the 2020 EverBatt model, developed by Argonne National Laboratory (52). The collected material recovery fractions and recycling inventory data are applied to assumed recycling scenarios in Europe, China, United States, Germany, and United Kingdom (using current and future electricity grid mixes). This sensitivity analysis shows what happens if the recycling is done in a country where the electricity is not as decarbonized, e.g. China, which dominates the current global recycling capacity; or if it is in Europe, then Germany, where grid are still heavily coal-dependent, and the United Kingdom, where the grid is less CO_2_ intensive compared to EU average.

We consider three recycling techniques as follows: pyrometallurgical recycling, hydrometallurgical recycling, and direct recycling as aligned in Everbatt (24). Each recycling technique has its unique characteristics and can recover specific components and materials of the LIB. We then evaluate the environmental impacts of recycling techniques for different cathode chemistries, based on different amount of recovered material. The battery cathode chemistries include NMC111, NMC532, NMC622, NMC811, NCA, and LFP. The amount of material assumed to be recovered from spent batteries through each of the recycling technologies for different battery chemistries is given in Table S22 in the supplementary information.

This study assumes two closed-loop recycling scenarios—European Battery Scenario and Circular Battery Scenario—and assumes that the selected countries adopt these scenarios. These scenarios are not predicting what will happen but will try to put into context how recycling could reduce impacts in the future. Secondary materials will never meet the rising demand for batteries, meaning that although recycling provides useful complementary resources, it can only provide a tiny fraction of the total demand (23). Table S24 summarizes the assumptions for the recycling scenarios.

- European Battery Scenario—Based on the mandatory minimum levels of recycled content declared by the new EU regulatory framework for batteries (27). Minimum levels of secondary materials would be set to 12% cobalt, 4% lithium, and 4% nickel for 2030; increasing to 20% cobalt, 10% lithium, and 12% nickel in 2035. Therefore, this scenario assumes that these shares of secondary materials in battery remanufacture while the remaining share will come from primary materials. Manganese, graphite, copper, and aluminum are assumed to be incorporated at 10%.
- Circular Battery Scenario—Most materials are secondary materials (recycled). This scenario assumes a complete closed loop, where the battery reaches end of life, and is replaced with secondary materials from recycling alongside supplements with primary materials at the same battery chemistry and the same capacity. The ratio of recycled materials included in secondary battery manufacturing is based on the efficiency of material recovery for different recycling technologies given in Table S21, e.g. lithium recovered via hydrometallurgy at 90% efficiency will include 10% primary lithium and 90% secondary lithium.

## Supplementary Material

pgad361_Supplementary_Data

## Data Availability

We use the ecoinvent and GREET database and process models to compile an environmental dataset for battery supply chain. All data presented in this paper are available from: https://doi.org/10.6084/m9.figshare.23454449.

## Supplementary Material

Figs. S1 to S9—supplementary pdf document.

Supplmentary Data Tables S1 to S28—https://doi.org/10.6084/m9.figshare.23454449.
