# Supplementary material for: Estimating the environmental impacts of global lithium-ion battery supply chain: A temporal, geographical, and technological perspective
Source: PNAS Nexus. 2023 Nov 28;2(11):pgad361. doi: 10.1093/pnasnexus/pgad361 (PMC10683946; doi:10.1093/pnasnexus/pgad361)
Supplement: pgad361_Supplementary_Data [file pgad361_supplementary_data.zip › 606466_0_supp_10458360_s37hcb_convrt.pdf]

PNAS nexus

## Supporting Information for

# Estimating the environmental impacts of global lithium-ion battery supply chain: a temporal, geographical, and technological perspective

Jorge A. Llamas-Orozco[1], Fanran Meng[2*], Gavin S. Walker[1], Amir F.N. Abdul-Manan[3], Heather L. MacLean[4], I. Daniel Posen[4], Jon McKechnie[1]

[1] Sustainable Process Technologies Research Group, Department of Mechanical, Materials and Manufacturing Engineering, Faculty of Engineering, University of Nottingham, Nottingham, NG7 2RD, United Kingdom

[2] Department of Chemical & Biological Engineering, Faculty of Engineering, Sir Robert Hadfield Building, Mappin Street, Sheffield, S1 3JD, United Kingdom

[3] Strategic Transport Analysis Team, Transport Technology R&D, Research & Development Center (R&D), Saudi Aramco, Dhahran, 31311, Saudi Arabia

[4] Department of Civil & Mineral Engineering, University of Toronto, 35 St. George Street, Toronto, Ontario, M5S 1A4, Canada

*Corresponding authors. Email: f.meng@sheffield.ac.uk; jon.mckechnie@nottingham.ac.uk

**This PDF file includes:**

Method for calculating the carbon emissions of electricity generation.
Global warming potential values.
Figure S1. Carbon intensity of electricity.
Figure S2. Cradle-to-gate primary energy demand for all chemistries.
Figure S3. NMC811 supply chain greenhouse gas emissions.
Figure S4. LFP supply chain greenhouse gas emissions.
Figure S5. Sensitivity of greenhouse gas emissions.
Figure S6. GHG emissions of NMC811 by decarbonising the electricity sector to 2050.
Figure S7. Battery market share by 2050.
Figure S8. GHG emissions of cathode production from virgin and secondary materials

**Calculation of the electricity generation carbon emission factor**

$$CO_2\text{/kWh for electricity generation} = \sum fuels \frac{(Input\ Energy\ source * EF\ fuel)}{(1 - T\&D\ losses)}$$

where:

- $CO_2$/kWh: carbon emissions (in CO2/kWh) calculated at the generation site.
- $\sum fuels$ : Sum over fuels which include coal, natural gas, oil, nuclear, hydro, biofuels, wind, solar pv, solar thermal, geothermal, tide, and waste.
- Input Energy source: Energy fuel source input, expressed in energy unit.
- EF fuel: default emission factors as provided in the ecoinvent database 3.7 2020.
- T&D losses: Total transmission and distribution losses in the grid. Note that data quality for electricity T&D losses may be very variable across countries.

## Electricity Mix

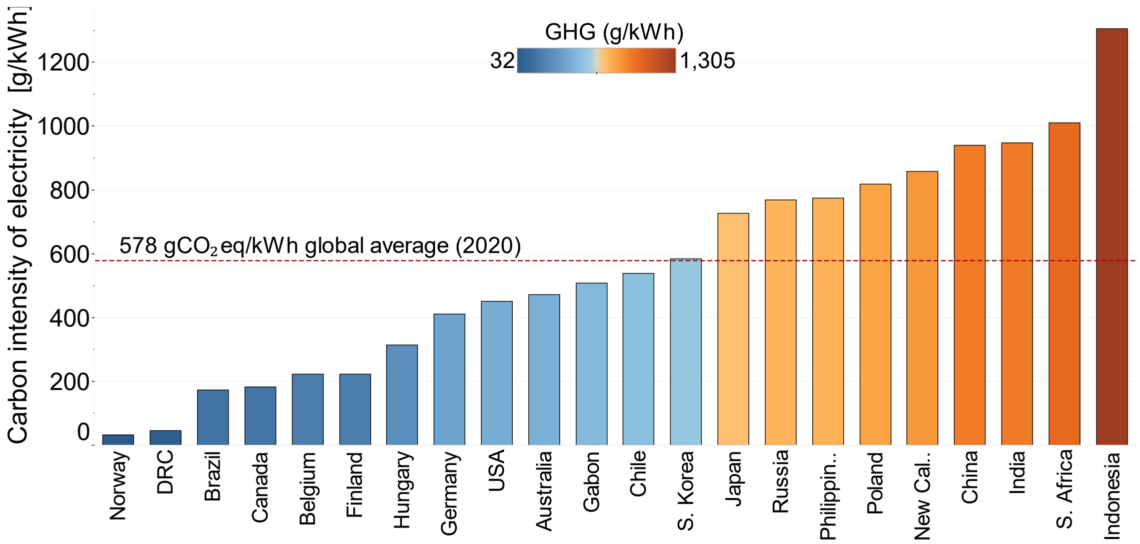


**Figure S 1.** Carbon intensity of electricity generation per country (2020). Detailed numerical data is presented in Table S5 in the supporting information.

## Total Battery Production

PED results are largely parallel the greenhouse gas emissions results: nickel-based cathode chemistries are more energy intensive than the LFP alternative, driven by the cathode active materials used in NCA and NMC chemistries. Detailed greenhouse gas emissions breakdown by material types for each LIB cathode is provided in **Table S10** in the SI.

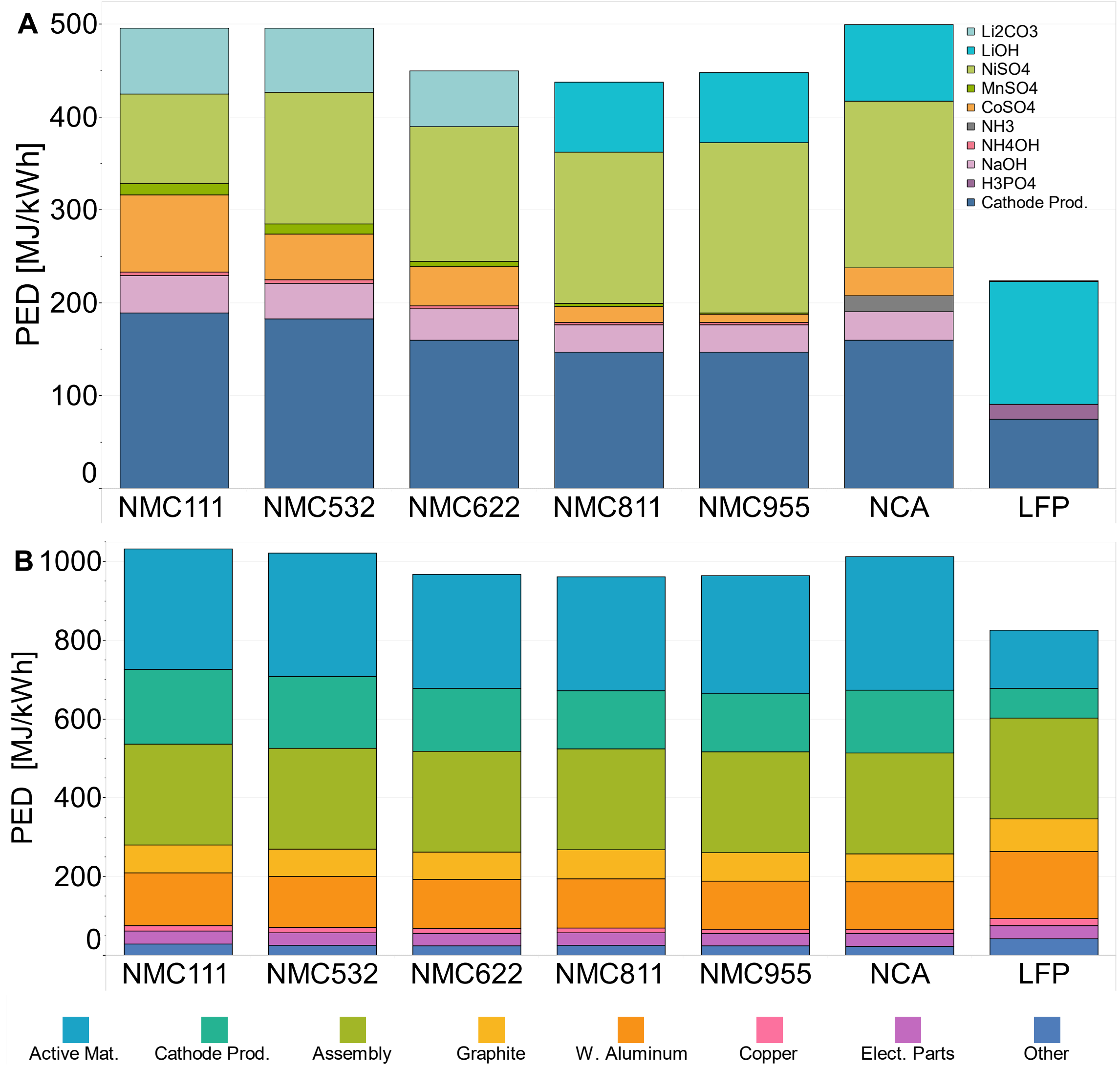


**Figure S 2.** Cradle-to-gate Primary Energy Demand of 1kWh of **(A)** cathode materials and cathode production process **(B)** total battery. Materials such as binder (polyvinylidene fluoride), electrolytes ($LiPF_6$, Ethylene Carbonate, Dimethyl Carbonate), plastics (polypropylene, polyethylene, polyethylene terephthalate), steel, thermal insulation, and coolant are grouped into "Other", because they each contribute less than 1% to the total greenhouse gas emissions. Numerical data can be found in Tables S10 and S11 in the supplementary information.

**Supply Chain environmental impacts**

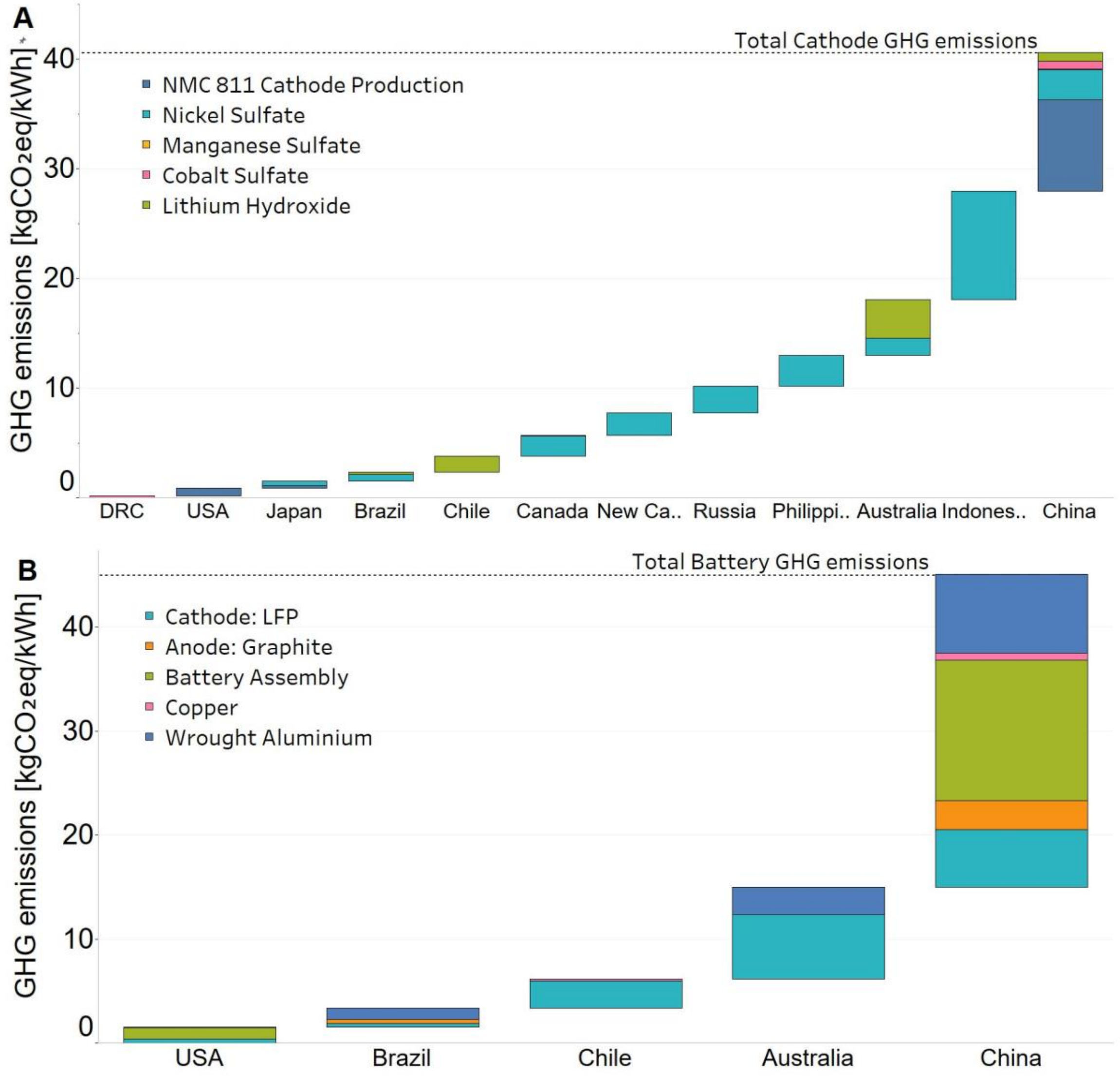


**Figure S 3.** NMC811 supply chain greenhouse gas emissions of **(A)** cathode active material - global production emissions of 45.47 $kgCO_2eq/kWh$. Sodium hydroxide (1.81 $kgCO_2eq/kWh$) and ammonium hydroxide (0.16 $kgCO_2eq/kWh$) are not included in the figure. Numerical values can be found in Table S12. **(B)** Total NMC811 battery production – global average production emissions of 79.6 $kgCO_2eq/kWh$. Numerical values can be found in Table S13. . The dashed horizontal line indicates the total GHG emissions of the cathode/battery, the floating charts indicate the relative emissions contributions by country.

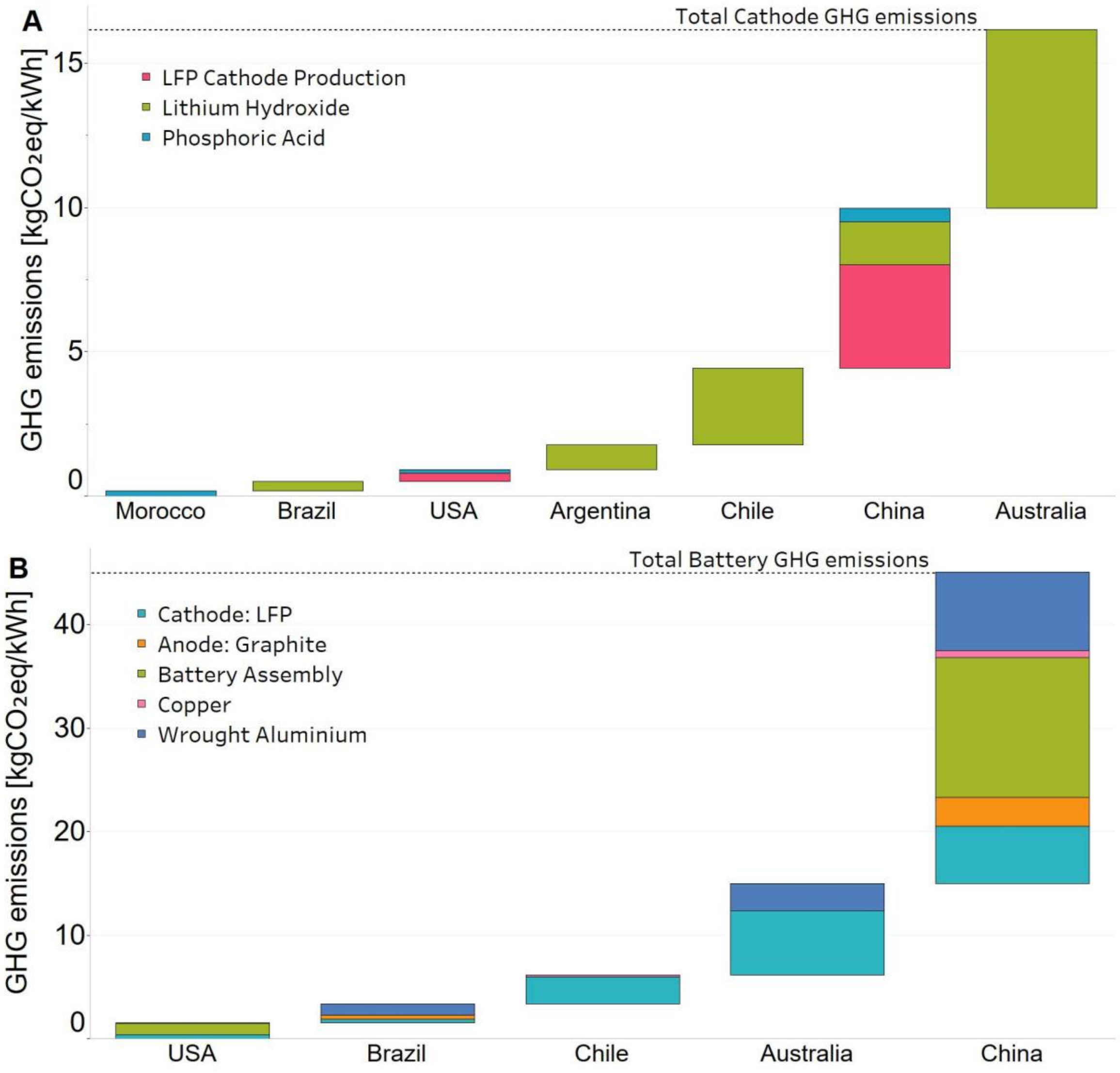


**Figure S 4.** LFP supply chain greenhouse gas emissions of **(A)** cathode active material - global production emissions of 17 kg$CO_2$eq/kWh. Numerical values can be found in Table S14. **(B)** Total LFP battery production - global production emissions of 56.4 kg$CO_2$eq/kWh. Numerical values can be found in Table S15. The dashed horizontal line indicates the total GHG emissions of the cathode/battery, the floating charts indicate the relative emissions contributions by country.

**Sensitivity GHG emissions**

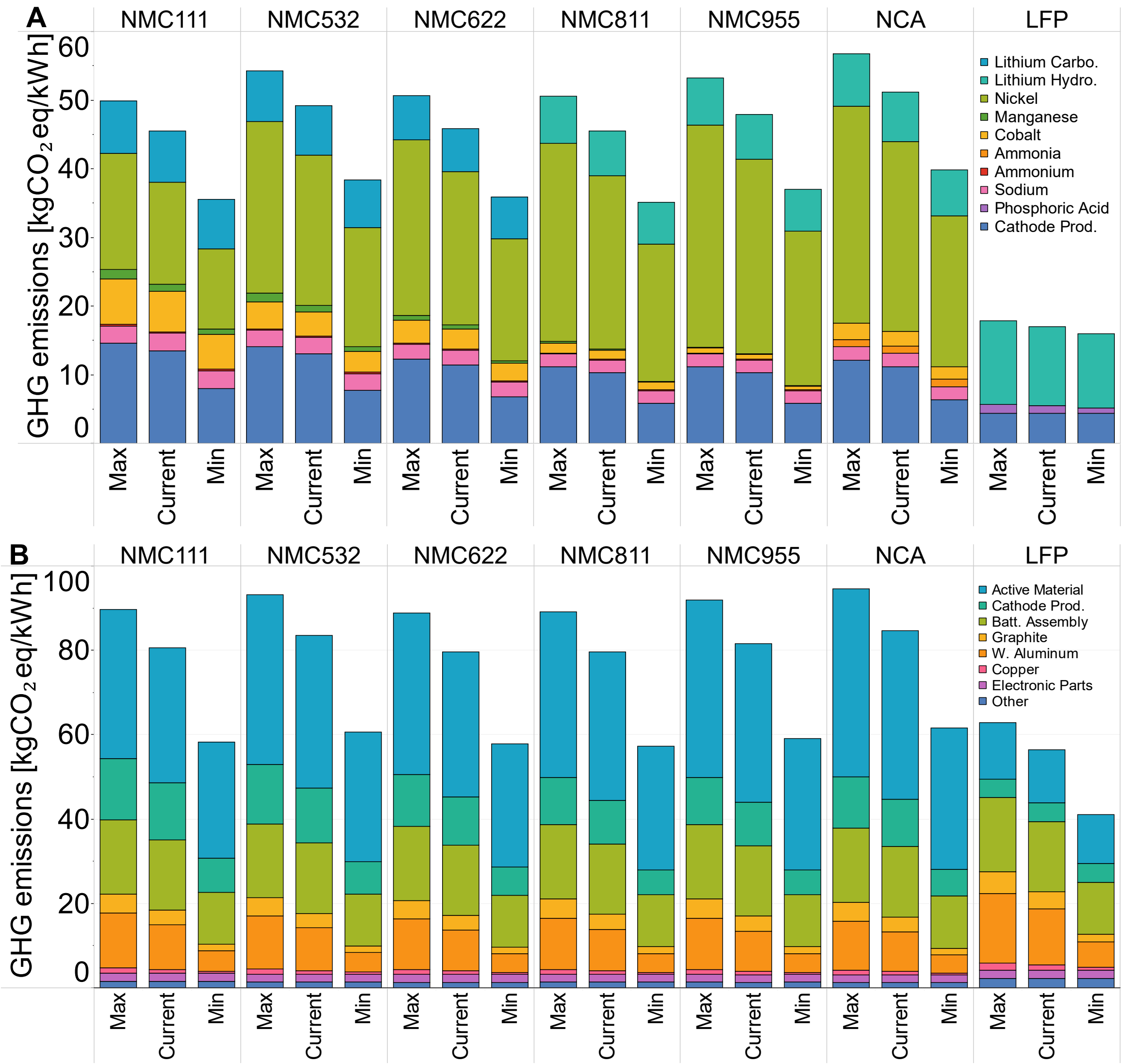


**Figure S 5.** Sensitivity greenhouse gas emissions for **(A)** Cathode production **(B)** Total battery production. Numerical data is available in Table S16 in the supplementary information.

**Key role of electricity decarbonization on future LIB production**

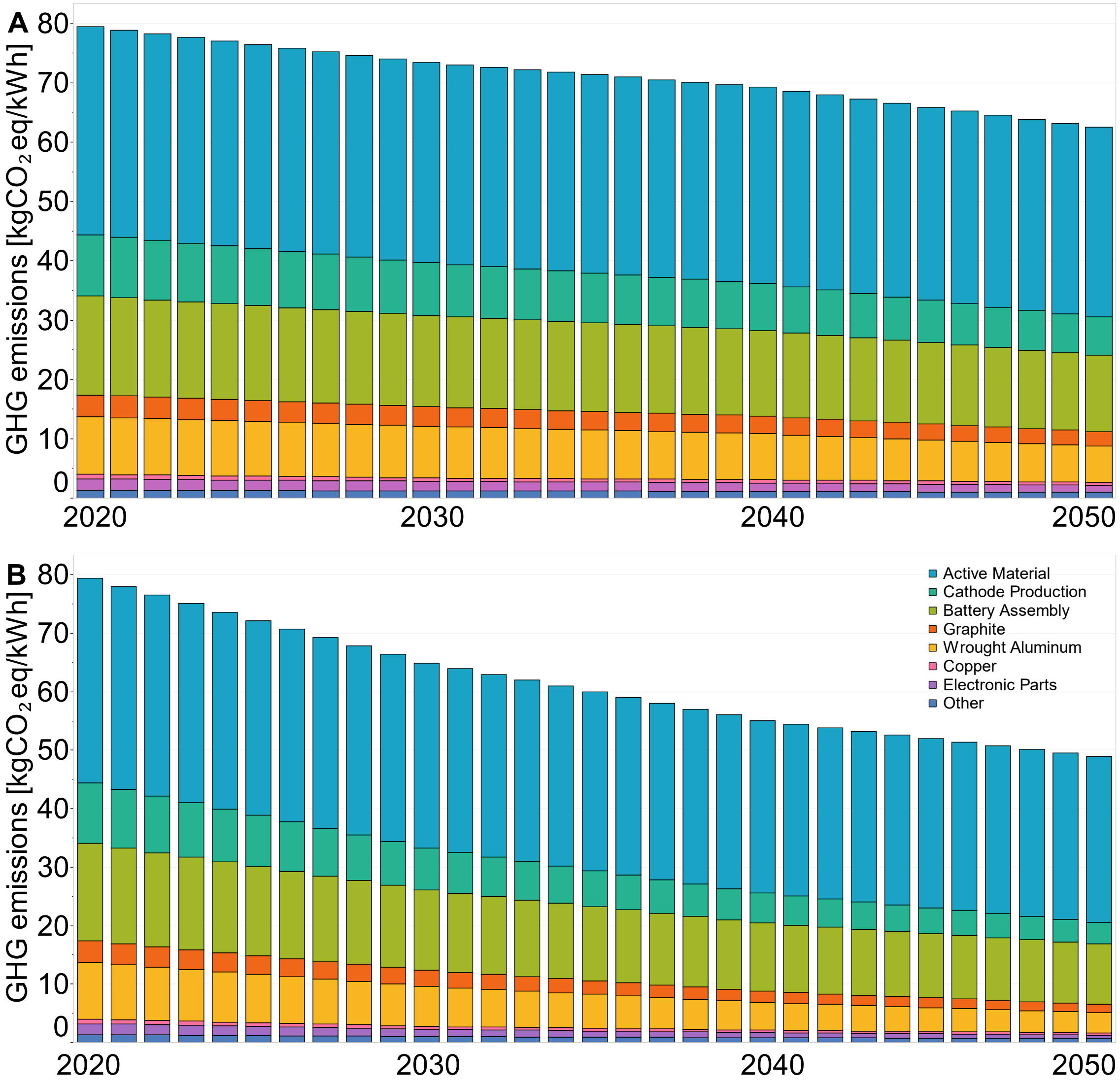


**Figure S 6.** Greenhouse gas emissions of NMC811 manufacturing by decarbonising the electricity sector to year 2050 in the SPS and SDS scenarios. **(A)** Stated Policies Scenario **(B)** Sustainable Development scenario. Detailed numerical data is presented in table S18 in the supplementary information.

**Future battery technology mix scenarios**

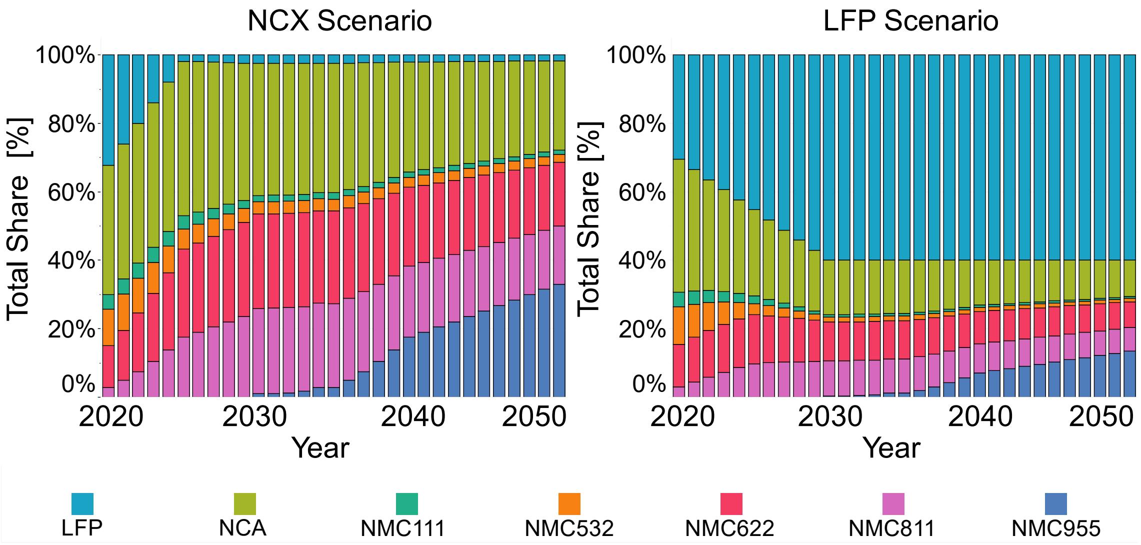


**Figure S 7.** Battery Market share by 2050. Detailed numerical data is presented in table S19 in the supplementary information.

**Primary and secondary impacts for cathode production**

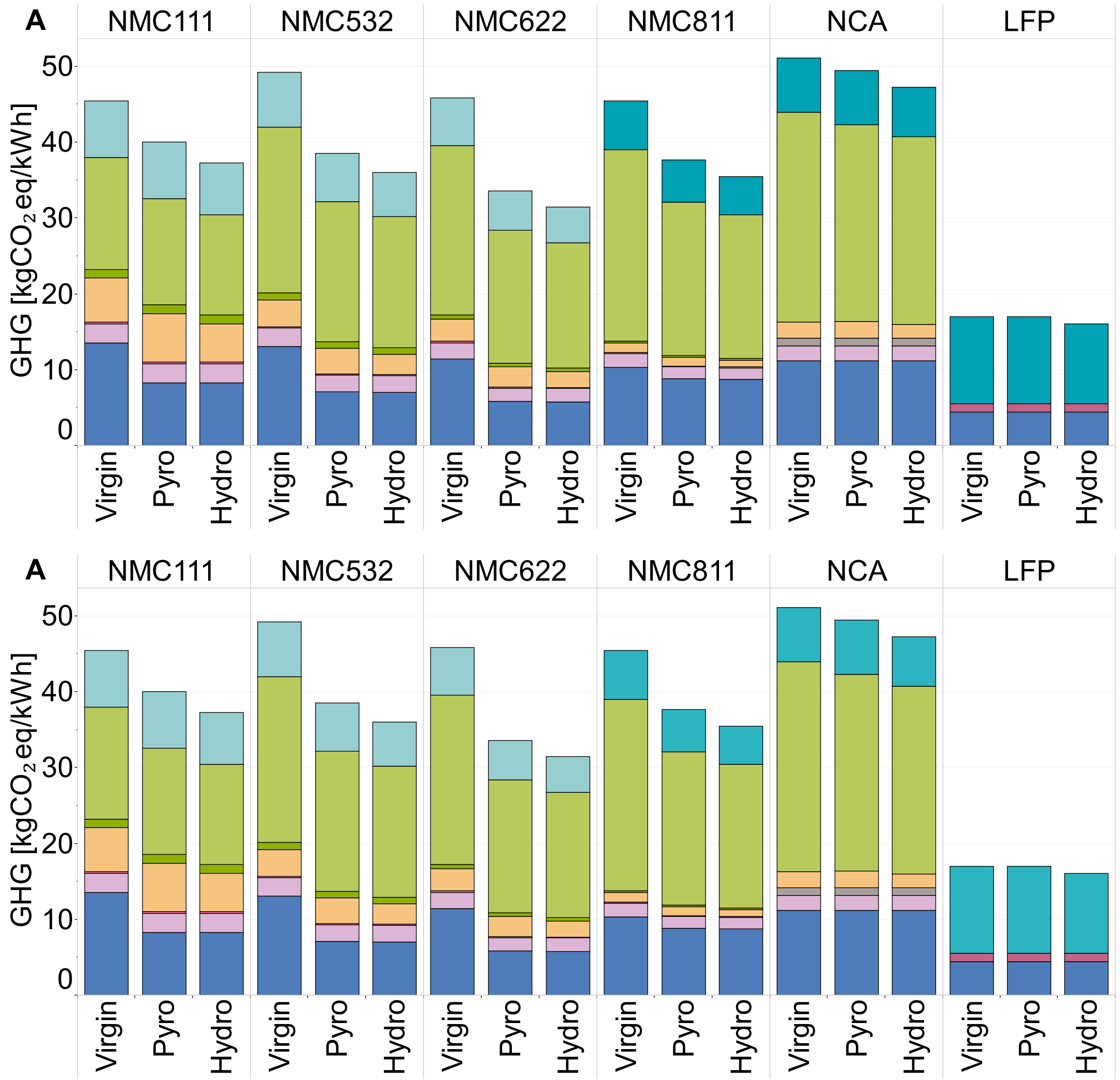


**Figure S 8.** Cathode production GHG emissions from virgin and secondary materials using different recycling technologies under **(A)** European Battery Scenario **(B)** Circular Battery Scenario. Detailed numerical data is presented in table S26 in the supplementary information.
